# FGF2 engineered SPIONs attenuate tumor stroma and potentiate the effect of chemotherapy in 3D heterospheroidal model of pancreatic tumor

**DOI:** 10.7150/ntno.38092

**Published:** 2020-01-01

**Authors:** Deby Fajar Mardhian, Aggelos Vrynas, Gert Storm, Ruchi Bansal, Jai Prakash

**Affiliations:** Targeted Therapeutics, Department of Biomaterials Science and Technology, Technical Medical Centre, Faculty of Science and technology, University of Twente, Enschede, The Netherlands.

**Keywords:** fibroblast growth factor 2, superparamagnetic iron oxide nanoparticles, pancreatic stellate cells, cancer-associated fibroblasts, pancreatic cancer

## Abstract

Pancreatic ductal adenocarcinoma (PDAC), characterized with abundant tumor stroma, is a highly malignant tumor with poor prognosis. The tumor stroma largely consists of cancer-associated fibroblasts (CAFs) and extracellular matrix (ECM), and is known to promote tumor growth and progression as well as acts as a barrier to chemotherapy. Inhibition of tumor stroma is highly crucial to induce the effect of chemotherapy. In this study, we delivered fibroblast growth factor 2 (FGF2) to human pancreatic stellate cells (hPSCs), the precursors of CAFs, using superparamagnetic iron oxide nanoparticles (SPIONs). FGF2 was covalently conjugated to functionalized PEGylated dextran-coated SPIONs. FGF2-SPIONs significantly reduced TGF-β induced hPSCs differentiation (α-SMA and collagen-1 expression) by inhibiting pSmad2/3 signaling and inducing ERK1/2 activity, as shown with western blot analysis. Then, we established a stroma-rich self-assembling 3D heterospheroid model by co-culturing PANC-1 and hPSCs in 3D environment. We found that FGF2-SPIONs treatment alone inhibited the tumor stroma-induced spheroid growth. In addition, they also potentiated the effect of gemcitabine, as shown by measuring the spheroid size and ATP content. These effects were attributed to the reduced expression of the hPSC activation and differentiation marker, α-SMA. Furthermore, to demonstrate an application of SPIONs, we applied an external magnetic field to spheroids while incubated with FGF2-SPIONs. This resulted in an enhanced effect of gemcitabine in our 3D model. In conclusion, this study presents a novel approach to target FGF2 to tumor stroma using SPIONs and thereby enhancing the effect of gemcitabine as demonstrated in the complex 3D tumor spheroid model.

## Introduction

Pancreatic ductal adenocarcinoma (PDAC) is one of the deadliest cancers with worst prognosis and a 5-year survival rate only of 8% 1. Annually, more than 50,000 people in U.S. and 90,000 people in Europe are diagnosed with PDAC 1. Most patients with PDAC are diagnosed when the tumor already reached the advanced stage and low survival is an irreparable outcome of late diagnosis that prevails in this cancer 2. In the advanced stage, the surgical treatment of pancreatic cancer is not feasible, making radiotherapy and chemotherapy the only option 3, 4. However, these treatments have only limited benefits with no enhanced clinical outcome 5, 6.

PDAC contains abundant tumor stroma which is comprised of cancer-associated fibroblasts (CAFs), endothelial cells, inflammatory immune cells, and extracellular matrix (ECM) 7. CAFs are the prominent cell types in the tumor stroma possessing large secretome of numerous growth factors and cytokines, which activate cancer cells and other cells in the tumor microenvironment 8-10. In addition, CAFs produce and remodel extracellular matrix (ECM), creating an impenetrable environment for chemotherapy and facilitating the formation of hypoxic and hypovascular network 9. The main precursor cells of CAFs in PDAC are pancreatic stellate cells (PSCs) 11. They are one of resident cells in the exocrine pancreas participating in the pathogenesis in major disorders, after transforming from quiescent state to activated state 12. Activated PSCs are therefore the most interesting target cell type for modulating the tumor stroma 11.

Activation and differentiation of PSCs into myofibroblastic CAFs is commonly regulated by Transforming growth factor beta (TGF-β) 13. In literature, fibroblast growth factor 2 (FGF2, 17.2 kDa) has been shown to exert inhibitory effects against TGF-β 14-18. The activation of extracellular-regulated kinase 1/2 (ERK1/2) pathway by FGF2 has been shown to counteract the TGF-β mediated activation in endothelial cells or fibroblasts 14-18. We therefore hypothesized that FGF2 could be used to antagonize the effect of TGF-β in PSC activation and thereby inhibit the tumor stroma induced pro-tumorigenic functions. However, use of biologics such as cytokines or growth factors is bound to several limitation including poor stability, rapid metabolism, and undesired side effects. In human body, the half-life of FGF2 is only about 7.6 hours 19, 20. To improve the half-life of such biological, protein engineering or nanoparticle conjugation may prove useful. Recently, we have reported the use of superparamagnetic iron oxide nanoparticles (SPIONs) to deliver a biologic, relaxin hormone, to tumor stroma and fibrotic liver 21, 22. SPIONs have high biocompatibility and large surface area with functional groups to conjugate a range of ligands and drugs, with high targeting and drug delivery efficiency 23, 24. SPIONs also have the ability to respond to the external stimuli such as magnetic field and heat, and have been used in clinics e.g. Ferumoxytol® 25-27. Surface conjugation of biological such as cytokines and growth factors to SPION may provide several benefits such as better pharmacokinetics, confinement of molecules on the surface allowing better interaction with the target receptor, and enhanced stability. Additionally, multivalent interaction may enhance the efficacy due to stronger interaction as achieved in our previous study with another molecule 21.

In this study, we aimed to deliver FGF2 using SPIONs to inhibit the activation of PSCs and thereby inhibit the stroma-induced tumor cell growth. We first investigated the target receptors of FGF2 in the TGF-β activated primary human PSCs (hPSCs). Then, we conjugated human recombinant FGF2 to PEGylated dextran-coated SPIONs using covalent conjugation chemistry. Next, we characterized FGF2-SPIONs to confirm the successful conjugation of FGF2 to SPIONs. Then, we examined the effect of FGF2-SPIONs on activation, migration and contraction of hPSCs. Finally, we developed stroma-rich 3D heterospheroids of pancreatic tumor cells (PANC-1) and hPSCs as a model representing fibrotic tumor stroma and examined the efficacy of FGF2-SPIONs with or without chemotherapy on the tumor spheroid growth with or without the presence of external magnetic field.

## Materials and Methods

### Cells

Primary human pancreatic stellate cells (hPSCs, ScienCell, Carlsbad, USA) were cultured in complete Stellate Cell medium (supplemented with 1% Stellate Cell Growth Supplements (SteCGS), 1% penicillin/ streptomycin, and 2% FBS) (ScienCell). PANC-1 pancreatic cancer cells were obtained from American Type Culture Collection (Manassas, USA) and were cultured in Dulbecco's Modified Eagles Medium (DMEM, Lonza, Verviers, Belgium) high glucose (4.5 g/l) with L-glutamine (GE Healthcare, Vienna, Austria) supplemented with 100 μg/ml penicillin/s treptomycin (Sigma Aldrich, St. Louis, USA), and 10% FBS (Lonza). Cells were maintained at 37 °C in a humidified 5% CO_2_ atmosphere. To minimize self-activation in hPSCs, we performed the experiments with early passage cells and only used them up to 8 passages.

### Quantitative real time PCR

To examine the expression of FGF receptors and differentiation/activation markers in TGF-β-mediated hPSC activation, cells were seeded into a 12 well plate at a seeding density of 50,000 cells/well. After 24h, cells were starved for 24 h, then treated with human recombinant TGF-β (Peprotech, Hamburg, Germany). After 24 h of the incubation, cells were lysed and total RNA were isolated using GenElute™ Mammalian Total RNA Miniprep Kit (Sigma Aldrich) and the RNA concentration was measured using a NanoDrop® ND-1000 Spectrophotometer (Thermo Scientific, Waltham, USA). cDNA was synthesized with iScript™ cDNA Synthesis Kit (BioRad, Veenendaal, The Netherlands), and 10 ng cDNA were used for each PCR reaction. The real-time PCR primers (Table 1) were purchased from Sigma Aldrich. Quantitative real time PCR was performed with 2x SensiMix SYBR and Fluoroscein Kit (Bioline GmbH, Luckenwalde, Germany) using a BioRad CFX384 Real-Time PCR detection systems (BioRad). Gene expression levels were normalized to the expression of the house-keeping gene 18s rRNA.

### F-actin staining

To analyse the morphological changes on F-actin organization in hPSCs, cells were seeded into 24 well plate at a seeding density of 4,000 cells/well and starved for 24 h. hPSCs were then incubated 5 ng/ml TGF-β for 24 h and then they were washed three times with DPBS (Lonza) and fixed in 4% formaldehyde (Sigma Aldrich) in DPBS for 20 minutes. After permeabilizing the cells with 0.1 M Triton X-100 (Sigma Aldrich) for 5 minutes, F-actin was stained with phalloidin (Life Technologies, Carlsbad, USA) at concentration of 250 ng/ml for 60 minutes. Next, cells were washed with DPBS and then mounted with DAPI (Sigma Aldrich) containing mounting medium. Images were captured using an EVOS FI fluorescent microscope (Life Technologies) at excitation/emission 385nm/460nm (DAPI) and excitation/emission 540nm/565nm (F-actin).

### FGF2 conjugation to SPIONs

The conjugation of FGF2 to SPIONs was performed chemically using carbodiimide chemistry. One hundred microliter SPIONs (nanomag®-D-spio, dextran coated, PEG-COOH functionalized, 20 nm) (Micromod, Rostock, Germany) at concentration 5 mg/ml were added with 50 μl mixture of 5 μM 1-ethyl-3-(3-dimethylaminopropil) carbodiimide (EDC, Sigma Aldrich) and 17.4 μM N-hydroxysuccinimide (NHS, Sigma Aldrich) dissolved in 2-(N-morpholino) ethanesulfonic (MES, Sigma Aldrich) buffer (pH 6.3). After 45 minutes of reaction with gentle shaking, SPIONs were washed with PBS and centrifuged in Amicon® Ultracel® 30 kDa centrifugal filter tubes (Merck, Kenilworth, USA) at 5000 RPM thrice to remove excess of salts and reagents. Eventually, the activated SPIONs were reacted with 15 µg FGF2 (MW 17.2 kDa) (Peprotech, NJ, USA) overnight at 4 °C with gentle shaking. The next day, the reaction was stopped by washing with PBS three times with Amicon® Ultracel® 30 kDa centrifugal filter tubes. The supernatant of the first washing was collected for dot blot analysis. To deactivate the remaining carboxylic group on the SPIONs, they were reacted with 10 µg glycine for 30 minutes at room temperature (RT). The recovered FGF2-SPIONs were washed with PBS three times in dialysis centrifuge tubes and resuspended in 100 µl PBS.

### Dot blot

To evaluate the yield of the conjugation, 5 µl of FGF2, SPIONs, FGF2-SPIONs at different dilutions and supernatant were spotted on nitrocellulose membrane and allowed to dry for 10 minutes. The membrane was then incubated in 5% non-fat dry milk dissolved in tris buffered saline with 0.1% Tween® 20 (TBST-20) (Thermo Scientific). After 1 hour incubation, the membrane was washed three times with TBST-20, followed by incubation with anti-FGF2 primary antibody (Cell Signaling Technology, Leiden, The Netherlands) and species-specific horseradish peroxidase (HRP) labelled secondary and tertiary antibody (DAKO, Glostrup, Denmark). FGF2 was detected with Pierce™ ECL Plus Western Blotting substrate kit (Thermo Scientific) and the membranes were exposed to FluorChem™ M System (ProteinSimple, San Jose, USA). Quantitative analyses were performed using ImageJ software (NIH, Bethesda, MD).

### Prussian blue staining

To ensure the recovery of nanoparticles, 5 µl of FGF2, SPIONs, FGF2-SPIONs, and supernatant were spotted on nitrocellulose membrane and allowed to dry for 10 minutes. Iron oxide was detected with Prussian Blue test kit (Sigma Aldrich) containing potassium ferocyanide and hydrochloric acid in 1:1 ratio. Image was captured using normal digital camera.

### Dynamic light scattering and zeta potential

To evaluate the resulted size of conjugation, 5 µl of SPIONs or FGF2-SPIONs were diluted in 1 ml PBS and loaded in polystyrene cuvette. To evaluate the change in zeta potential, 5 µl of SPIONs or FGF2-SPIONs were diluted in 1 ml KCl 10 mM and loaded in folded capillary cells DTS1060 for zeta potential measurement (Malvern Panalytical). Examination of size or zeta potential of nanoparticles were performed with Zetasizer Nano ZS (Malvern Panalytical, Malvern, UK). Assays were performed with at least 2 mins equilibration and minimum 15 runs per measurement.

### Binding/uptake of FGF2-SPION

To evaluate the affinity of FGF2-SPION to hPSCs, cells were seeded into 24 well plate at a seeding density of 4,000 cells/well and starved for 24 h. hPSCs were then incubated with 5 ng/ml TGF-β for 24 h and then they were washed three times with PBS. Next, hPSCs were incubated with SPION or FGF2-SPION in DPBS (Lonza) with 2 mM Mn^2+^ (Sigma) and 2 mM Ca^2+^ (Sigma) at RT for 1 h or 2 h. As controls, non- TGF-β treated hPSCs were incubated with SPION or FGF2-SPION at RT for 1 h. Cells were then washed thrice with DPBS and fixed with 4% formaldehyde in DPBS for 15 mins. After fixation, cells were washed with thrice with DPBS. Prussian blue iron staining kit containing potassium ferocyanide and hydrochloric acid in 1:1 ratio (Sigma Aldrich) was applied in each well and let at RT for 30 mins. Finally, the cells were washed with demi water and mounted with Aquatex mounting medium (Sigma Aldrich). Images were made the next day with Nikon E400 microscope (Nikon, Tokyo, Japan). Quantitative analyses were performed using ImageJ software (NIH, Bethesda, MD).

### Immunofluorescent staining

hPSCs were seeded into 24 well plates at a seeding density of 10,000 cells/well. After overnight, cells were starved for 24 h and treated with 5 ng/ml TGF-β and FGF2 at 250 ng/ml (low) or 500 ng/ml (high). After 48 h incubation, cells were fixed with acetone:methanol (1:1) for 30 minutes at -20 °C followed by drying at RT and rehydration with PBS. To analyse protein expression of α-SMA and collagen-1, cells were incubated with respective primary antibody for 1 h at room temperature followed by fluorescence labeled secondary antibody for 30 minutes in dark condition. Finally, cells were mounted with DAPI-containing fluoroshield (Sigma Aldrich). Images were made using an EVOS FI fluorescent microscope (Life Technologies) at excitation/emission 385nm/460nm (DAPI), excitation/emission 485nm/530nm (GFP) and excitation/emission 590nm/617nm (RFP). Images were analysed using ImageJ software (NIH).

### Western blot analysis

To evaluate the effect of FGF2 on the protein expression of hPSC activation markers, hPSCs were seeded into a 12 well plate at seeding density 50,000 cells/well. After the overnight incubation, the medium was changed with 0% FBS Stellate Cell medium to starve hPSCs for 24 h. Subsequently, hPSCs were incubated with 5 ng/ml TGF-β1 and FGF2, SPIONs, or FGF2-SPIONs (equivalent to 250 ng/ml or 500 ng/ml FGF2) for 24 h to examine the effect on protein expression of different markers such as α-SMA and collagen-1, and phosphorylation of Smad2 and ERK1/2. Cells were lysed using 1x blue loading buffer containing 1x DTT reducing agent (Cell Signaling Technology) and homogenized using ultrasonication. Protein lysates were separated on a 4-20% Tris-Glycine gel (Thermo Scientific) and then transferred onto a PVDF membrane (Thermo Scientific). The blots were incubated with the primary antibody overnight at 4°C followed by incubations with species specific HRP conjugated secondary antibody for 1 h at room temperature. Proteins were detected with Pierce™ ECL Plus Western Blotting substrate kit (Thermo Scientific) and the membranes were exposed to FluorChem™ M System (ProteinSimple, San Jose, USA). The protein signals were quantified using ImageJ Software (NIH) and the target protein expression levels were normalized to β-actin.

### Wound healing assay

To evaluate the effect of FGF2 and FGF2-SPIONs on hPSCs migratory property, cells were seeded into a 24 well plate at a seeding density of 60,000 cells/well and starved for 24 h. After starvation, a scratch was made on the culture plate using a 200 μl pipette tip fixed in a custom-made holder. Cells were washed with serum-free media and incubated with serum-free media and FGF2 or FGF2-SPIONs (equivalent to 250 ng/ml FGF2). Images were captured at t=0h and t=12h using an EVOS microscope. Images were analyzed using ImageJ software to quantitate the area of the scratch and represented as percentage of wound closure.

### 3D collagen gel contraction assay

A collagen suspension (5 ml) containing 3.0 ml collagen G1 (5 mg/ml, Matrix biosciences, Morlenbach, Germany), 0.5 ml 10x M199 medium (Sigma), 85 ul 1N NaOH (Sigma) and sterile water was mixed with 1.0 ml (2 × 10^6^ cells) hPSCs. Collagen gel-cells suspension (0.6 ml/well) was plated in a 24-well culture plate and allowed to set for 1 h at 37 °C. Once set, gels were detached from the culture wells and 1 ml of serum free medium was added with or without TGF-β (5 ng/ml). To study the effect of FGF2 and FGF2-SPIONs, 1 ml of serum free medium with TGFβ (5 ng/ml) and FGF2 or FGF2-SPIONs (equivalent to 250 ng/ml FGF2) was added to the detached gels. Representative images were made at 96 h using a normal digital camera. Measurement of collagen gel diameter was performed using ImageJ (NIH).

### Spheroid formation

3D heterospheroids containing mixture of PANC-1 cancer cells and hPSCs were prepared in 96 round bottom suspension well plate (Greiner Bio-One, Kremsmunster, Austria). Ninety-six round bottom suspension well plates were coated with 1% (w/v) Pluronic® F127 (Thermo Scientific) overnight and washed 2 times with sterile water before the seeding of cells mixture. hPSC and PANC-1 were trypsinized and suspended in their respected growth medium to a density 60,000 cells/ml. The hPSC and PANC-1 suspension were mixed 1:1 (v/v). To form spheroids, 100 µl cell suspension containing 6000 cells were seeded into a well. After 3 days, the spheroids were imaged using EVOS inverted microscope and treated with SPIONs, FGF2, or FGF2-SPIONs (equivalent to 250 ng/ml FGF2) with or without gemcitabine (3 µg/ml). Imaging and treatments were repeated after additional 3 days. After grown for total 9 days, spheroids were moved separately to a single well of a 96 well plate. CellTiter-Glo® 3D reagent (ProMega, Leiden, The Netherlands) was added to each well and after 30 minutes the luminescence signal was measured using bioluminescent reader (Varioskan LUX, Thermo Scientific). To analyze the protein expression in the spheroids using western blot technique, ten spheroids per group were collected and washed 3 times by decantation with DPBS. Spheroids were then lysed using 1x blue loading buffer containing 1x DTT reducing agent (Cell Signaling Technology) and homogenized using ultrasonication. Protein lysates were then proceed for western blot analyses.

### Graphs and statistical analyses

All graphs were made using GraphPad Prism version 5 (GraphPad Software Inc., San Diego, CA). All values are expressed as a mean + standard error of the mean (SEM). Statistical significance of the results was performed by a two-tailed unpaired student's t-test for comparison of two treatment groups. Differences were considered significant for a p-value of *p < 0.05, **p < 0.01, ***p < 0.001 respectively.

## Results

### FGF receptor 3c (FGFR3c) expression was induced in TGF-β activated hPSCs

**Figure 1:**
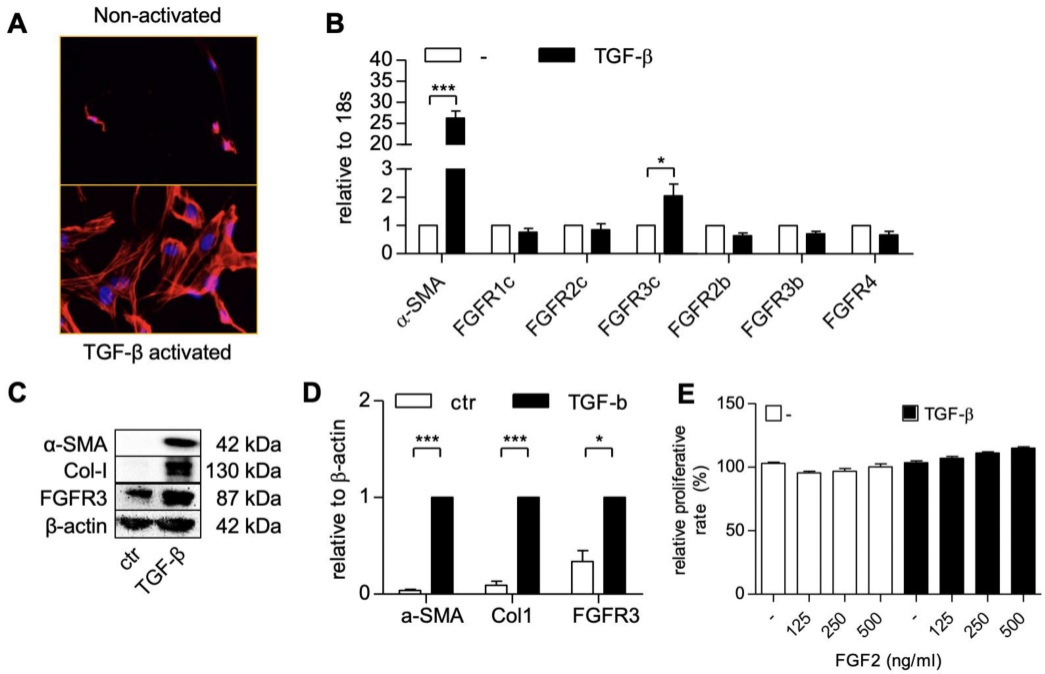
** hPSCs activation and the expression of the human fibroblast growth factor 2 (FGF2) receptors in hPSC.** F-actin staining (A) showing morphological changes in hPSCs after treated with 5 ng/ml TGF-β for 24h. Gene expression of α-SMA and FGFR-1c, -2c, -3c, -2b, -3b, -4. (B) in hPSCs after treated with 5 ng/ml TGF-β for 24h. Western blot (C) and quantification (D) showing protein expression of α-SMA, col1, and FGFR3 after treated with 5 ng/ml TGF-β for 48h. (E) Relative growth of cells after 48 hours treatment with FGF2 at different concentration and with or without TGF-β indicating no toxic effect exhibited by FGF2 at mentioned concentration. Data represents mean + SEM for at least 3 independent experiments. Statistical differences are *p<.05, ***p<.001.

We first activated patient-derived primary PSCs with TGF-β1 and examined the expression of different FGF receptor forms (FGFR-1c, -2c, 3c, 2b, -2b, -3b, -4) in the non-activated and activated hPSCs, using gene expression analysis. The activation and differentiation of hPSCs was confirmed by the morphological changes i.e. stretched and elongated shape, as shown with F-actin staining (**Figure 1A**) and the activation gene, α-SMA (**Figure 1B**). We found that all the tested forms of FGF receptor was expressed on the non-activated cells (**Figure 1B**). Interestingly, only FGFR3c was significantly induced after TGF-β1 treatment as compared to the non-activated hPSCs, as shown in **Figure 1B**. The upregulation of FGFR3 was also confirmed at protein level as shown with western blot in **Figure 1C** and **Figure 1D**.

### Preparation and characterization of FGF2-SPION conjugate

**Figure 2:**
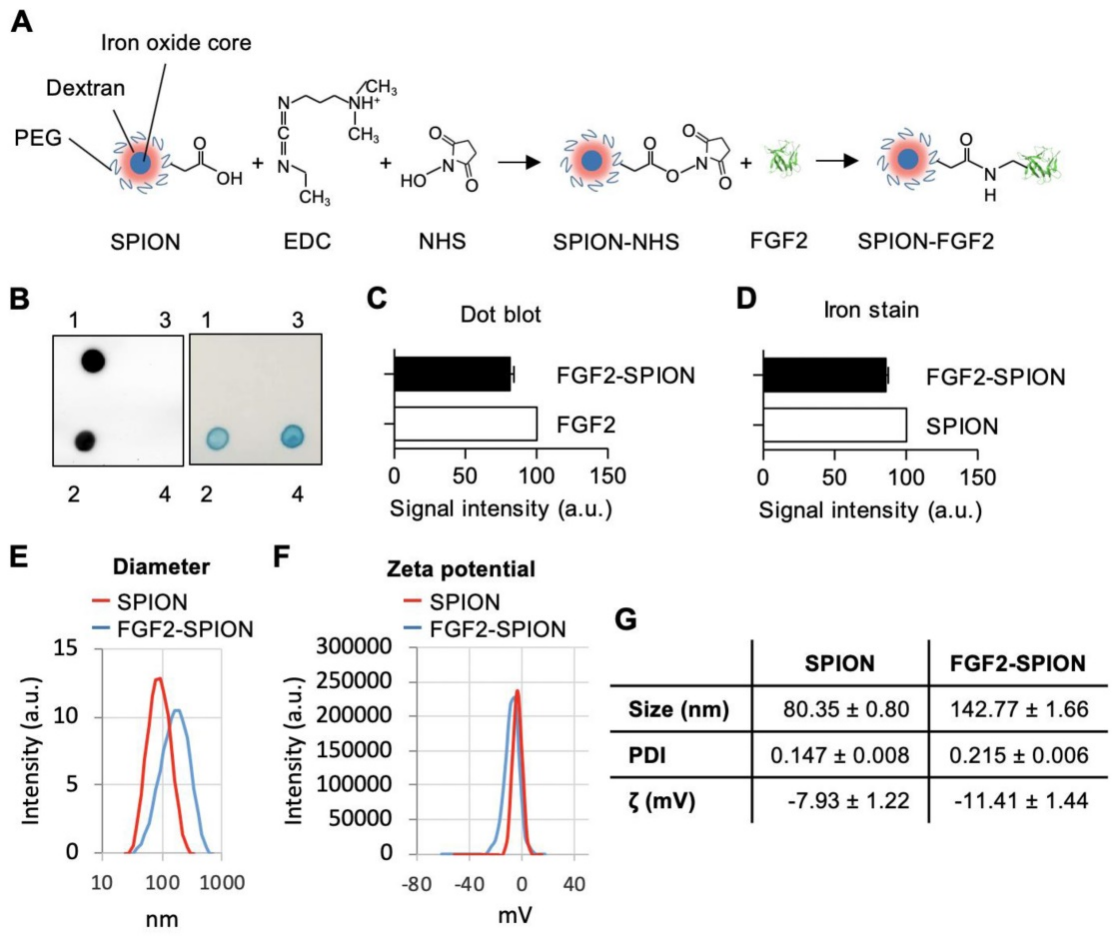
** Preparation and characterization of FGF2-SPION.** (A) Schematic representation of conjugation of FGF2 to SPION using carbodiimide chemistry. Immunoblot and iron staining (B) and quantification of FGF2 (C) and iron (D) showing successful conjugation and recovery. Labels 1-4 denote FGF2, FGF2-SPIONs, supernatant and SPIONs, respectively. Histograms of dynamic light scattering (E), zeta potential (F), and detailed physiochemical data (G) for SPION and FGF2-SPION. Data represents mean + SEM for at least 3 independent synthesis.

We conjugated FGF2 to SPIONs (dextran coated, PEG-COOH functionalized) (~20 nm) using carbodiimide reaction, as illustrated in a schematic representation in **Figure 2A**. SPIONs were reacted with FGF2 at molar ratio 1:5. The successful conjugation of FGF2 to SPIONs was confirmed with dot blot analysis by analyzing the expression of conjugated FGF2 using anti-FGF2 antibody followed by Prussian blue iron staining to detect iron oxide nanoparticles. As shown in the left panel of **Figure 2B**, FGF2 was present in the FGF2-SPIONs samples but was not detected in the supernatant or SPIONs samples, indicating that FGF2 was successfully conjugated to SPIONs and there was no unbound FGF2 in the supernatant. Quantitative analysis (**Figure 2C**) of FGF2 and FGF2-SPIONs indicate successful conjugation recovering about 81.2% of the added FGF2. In the right panel of **Figure 2B**, SPION was shown in the FGF2-SPIONs samples and was not detected in the supernatant. Quantitative analysis (D) of the iron stains showed that 85.5% SPIONs were recovered after conjugation likely due to loss during washing and purification steps. This resulted in 94.9% conjugation of FGF2 to SPIONs or approximately 5 FGF2 molecules per SPION. Dynamic light scattering (DLS) analysis showed increase in the hydrodynamic size of SPIONs after conjugation with FGF2 (**Figure 2E, 2G**). Next, we observed an increase of negative surface changes (zeta potential) of the particles after FGF2 conjugation (**Figure 2F, 2G**), indicating a successful conjugation.

### Binding of FGF2-SPIONs to activated hPSCs

**Figure 3:**
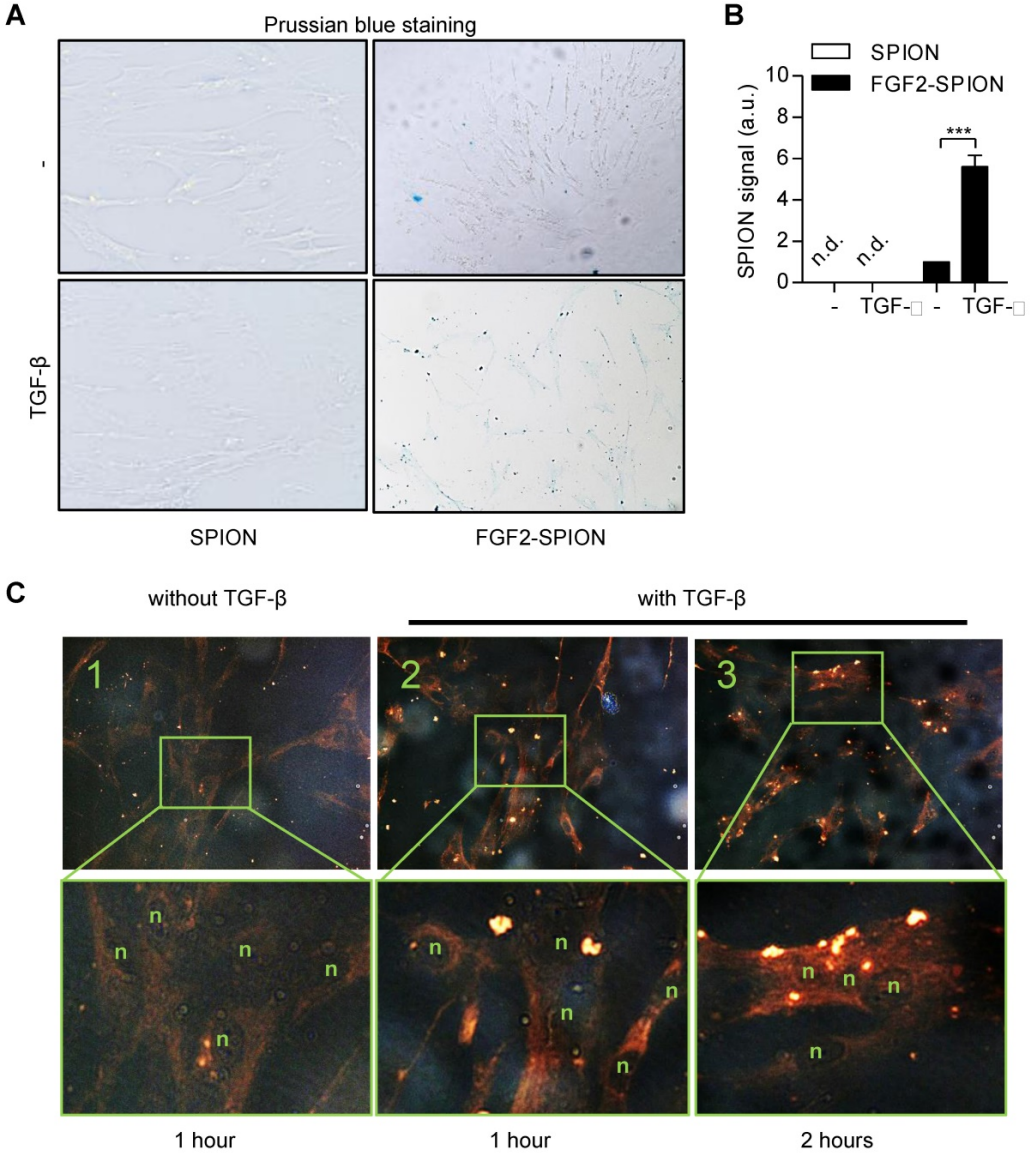
**Binding of FGF2-SPION on** non-activated **hPSCs and activated hPSCs.** Microscopic images (A) and quantitation (B) showing Prussian blue staining to detect iron oxide in hPSCs incubated with SPIONs or FGF2-SPIONs on non-activated hPSCs and TGF-β activated hPSCs. Representative images (C) of non-activated hPSCs (1) and hPSCs treated with 5 ng/ml TGF-β for 24 h (2, 3) and incubated with FGF2-SPION showing uptake of nanoparticles at 2 h incubation. (n) indicates nuclei. Data represents mean + SEM for at least 3 independent experiments. Statistical difference is ***p<0.001.

After preparing FGF2-SPIONs, we examined the binding of FGF2-SPIONs to hPSCs. We first activated hPSCs with 5 ng/ml TGF-β1 and after 24 h, we examined the binding of FGF2-SPIONs to hPSCs by incubating for 1 h or 2 h and then detecting SPIONs with Prussian blue staining. We found that the SPIONs alone did not show any binding to non-activated or activated hPSCs. FGF2-SPIONs, on the other hand, showed a weak binding to non-activated hPSCs (**Figure 3A**), and a strong binding to the activated hPSCs, as shown in **Figure 3B**. These data clearly indicate that the FGF2-SPIONs were able to bind to TGF-β activated hPSCs, most probably, via interaction with TGF-β induced FGFR3c. Further incubation time of FGF2-SPION in activated hPSCs has resulted in uptake of the nanoparticles as shown in **Figure 3C**.

### FGF2-SPIONs suppressed TGF-β induced hPSCs activation via Smad2/3 and ERK1/2 signaling pathways

**Figure 4:**
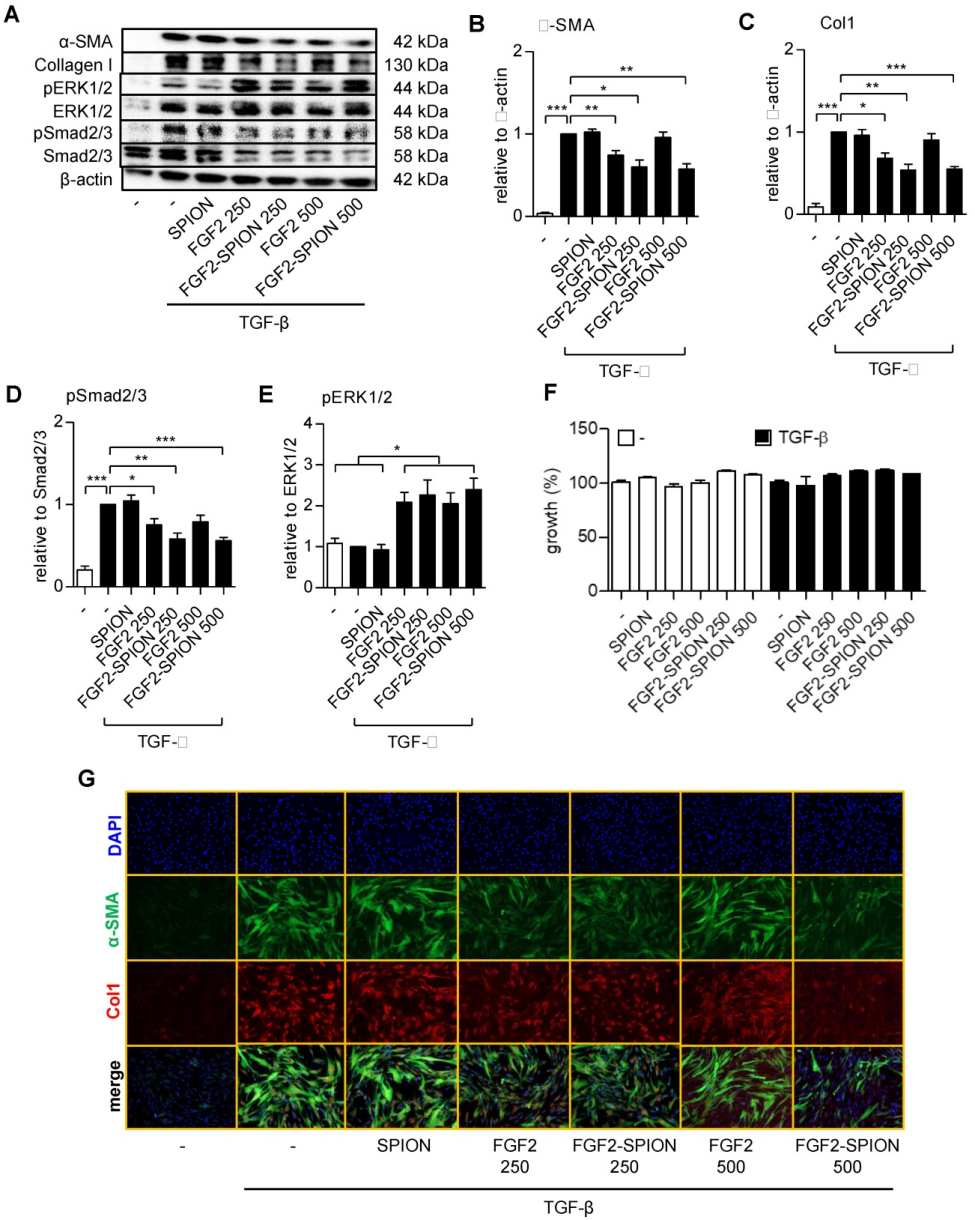
**Effect of FGF2-SPION on the differentiation of hPSCs.** Western blot (A) and quantitation showing the effect of FGF2 and FGF2-SPIONs at 250 ng/ml and 500 ng/ml on the protein expression of α-SMA (B), collagen-1 (col-1) (C) in hPSCs activated with 5 ng/ml TGF-β for 48 h compared to untreated hPSCs. Western blot and quantification showing the effect of FGF2 and FGF2-SPIONs on the phosphorylation of Smad2/3 (D) and ERK1/2 (E) in hPSCs activated with 5 ng/ml TGF-β for 1 h compared to untreated hPSCs. The protein expression levels for α-SMA and col-1 were normalized to β-actin, while pSmad2/3 and pERK1/2 were normalized to respective total protein levels. (F) Relative % growth of cells after 48 hours treatment with SPION, FGF2, or FGF2 SPION at concentration equal to 250 ng/ml or 500 ng/ml FGF2 and with or without TGF-β indicating no toxic effect exhibited by nanoparticles. (G) Representative immunofluorescence images showing the effect of FGF2 and FGF2-SPION on the protein expression of α-SMA and col-1 in TGF-β-activated hPSCs. Data represents mean + SEM for at least 3 independent experiments. Statistical differences are *p<0.05, **p<0.01, ***p<0.001.

To find out whether FGF2-SPIONs are pharmacologically active and inhibits the activation of hPSCs, we treated the cells with TGF-β and different concentrations of FGF2 (250 and 500 ng/ml) or FGF2-SPIONs (equivalent to 250 and 500 ng/ml FGF2). We chose these concentrations based on literature in which the receptor phosphorylation of FGFR3 by FGF2 was shown to be occurred in the range of 125-500 ng/ml in HEK293 cells 28. We found that FGF2 at 250 ng/ml reduced the activation of hPSCs, as shown with the expression of α-SMA and collagen 1α1 (col-1) (**Figure 4A-C**). However, surprisingly, the effects disappeared at higher concentrations (500 ng/ml), as shown with the western blot. Such a biphasic effect of FGF2 has also been reported 29-32. Interestingly, treatment with FGF2-SPIONs showed an inhibition of both α-SMA and col-1 at both 250 and 500 ng/ml, as shown with Western blot analysis and immunofluorescence staining (**Figure 4A-C, 4G**). At this range of concentration, FGF2, SPION, or FGF2-SPION showed no significant effect to the growth of hPSCs (**Figure 4F**). Furthermore, we evaluated whether FGF2-SPIONs inhibits the downstream signaling of TGF-β i.e. the phosphorylation of Smad2/3 and by induction of ERK1/2 signaling. We therefore examined the protein expression of phosphorylated Smad2/3 (pSmad2/3) compared to total Smad2/3 and phosphorylated ERK1/2 (pERK1/2) compared to total ERK1/2 using western blot analysis. The quantitative data confirmed that treatment with FGF2 inhibited the TGF-β-induced pSmad2 pathway (**Figure 4A, 4D**). As expected, TGF-β did not induce the signaling of pERK1/2 but importantly treatment with FGF2 or FGF2-SPIONs activated this pathway (**Figure 4A, 4E**). Notably, in contrast to TGF-β related pathways or markers, the effect of free FGF2 on ERK1/2 pathway was the same at the concentrations 250 and 500 ng/ml. These data clearly demonstrate that conjugation of FGF2 to SPIONs leads to not only retention of the effects of FGF2 but also entails to improved effects in inhibiting hPSCs activation.

### FGF2-SPIONs inhibited the migration and contraction of hPSCs

**Figure 5:**
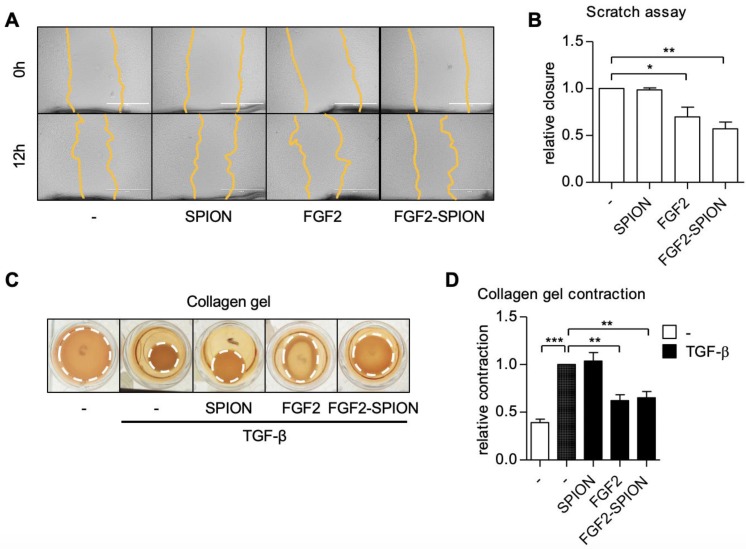
**Effect of FGF2-SPION on hPSCs migration and contractility.** Representative microscopic images (A) and quantification (B) showing the effect FGF2 (250 ng/ml) and FGF2-SPIONs (equivalent to 250 ng/ml FGF2) on the migration of hPSCs after 12 h of incubation. Representative images (C) and quantitation (D) showing the effect of FGF2 (250 ng/ml) and FGF2-SPIONs (equivalent to 250 ng/ml FGF2) on the hPSCs contraction in collagen 3D gel after 96 h incubation with 5 ng/ml TGF-β compared to untreated 3D collagen gel with hPSCs. Data represents mean + SEM for at least 3 independent experiments. Statistical differences are *p<0.05, **p<0.01, ***p<0.001.

PSCs are known to migrate to the invasive site as well as contract ECM in the tumor microenvironment leading to high stiffness 11. We therefore examined the effect of FGF2-SPIONs on the hPSCs migration using scratch assay and TGF-β-induced contraction of hPSCs in 3D collagen gel. Treatment with FGF2 or FGF2-SPIONs reduced the migratory property of hPSCs as shown with scratch assay (**Figure 5A-B**). Furthermore, we found that both FGF2 and FGF2-SPIONs inhibited the TGF-β-induced contractility of hPSCs, as shown with 3D collagen gel contraction model (**Figure 5C-D**).

Of note, unmodified SPIONs did not show any effect on migration and contraction of hPSCs. These data are in line with the effect of FGF2-SPIONs on α-SMA expression, the cytoskeletal marker. Altogether these results suggested that FGF2-SPIONs are able to counteract the differentiation of hPSCs into myofibroblastic CAFs and inhibit their functions.

### FGF2-SPIONs inhibited the stroma-induced tumor growth in 3D tumor spheroids

**Figure 6:**
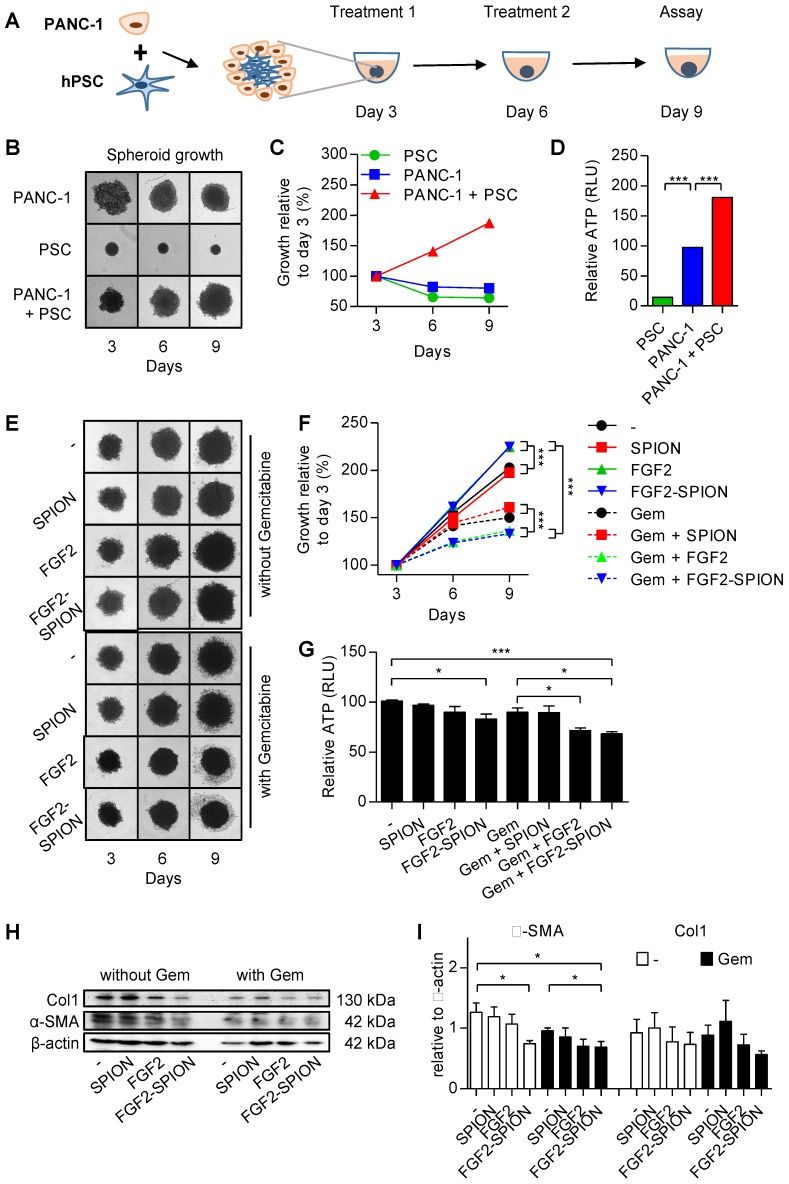
** Effect of FGF2-SPIONs on the tumor stroma and gemcitabine efficacy in 3D heterospheroids.** (A) Schematic representation of heterospheroid culture combining PANC-1 cells with hPSCs in a round bottom 96-well plate. Representative images (B) and quantification (C) showing growth of spheroids after spheroid formation at day 3 of the cell seeding. (D) Relative ATP content (%) at day 9 in homospheroids of PANC-1 or hPSCs and heterospheroids (PANC-1 + hPSCs). Representative images (E) and quantification (F) of PANC-1 + hPSC heterospheroids co-treated with gemcitabine and SPIONs or FGF2 or FGF2-SPIONs. (G) Relative ATP content (%) at day 9 shows the comparative ATP levels versus control untreated heterospheroids. Images were captured every 3rd day. Western blot (H) and quantitation (I) showing a reduction in α-SMA and col1 expression levels. Data represents mean + SEM for at least 3 independent experiments. Statistical differences are *p<0.05, **p<0.0.01, ***p<0.001.

To investigate the effect of FGF2-SPIONs on the tumor stroma (CAFs)-induced effect on the tumor growth in a complex 3D microenvironment *in vitro*, we established a 3D heterospheroid tumor model by co-culturing PANC-1 pancreatic tumor cells and hPSCs, as illustrated in **Figure 6A**. We first examined whether co-culturing of hPSCS with PANC-1 tumor cells induce the tumor growth. Therefore, we first compared the growth of homospheroids of PANC-1 with heterospheroids of PANC-1 and hPSCs. We found that until day 3 heterospheroids shrunk compared to homospheroids, which is likely due to the contractility of hPSCs as seen in hPSC alone spheroids (**Figure 6B**). Importantly, from day 3 onwards the PANC-1 + hPSC heterospheroids showed an increase in size compared to only tumor cell or hPSC homospheroids (**Figure 6B-C**). In addition to increased size of spheroids, heterospheroids resulted in increased ATP activity compared to homospheroids (**Figure 6D**). These data show that heterospheroids is the right model to investigate the effect on tumor stroma induced tumor growth.

After the tumor spheroids were established, we treated the tumor spheroids with FGF2 or FGF2-SPIONs (equivalent to 250 ng/ml FGF2) and standard chemotherapy gemcitabine (3 µg/ml) every 3 day. After 9 days of culturing, spheroids were collected and examined for their viability using ATP determination assay. Surprisingly, treatment with FGF2 or FGF2 SPION resulted in an increased size of heterospheroids (**Figure 6E-F**). This is most probably a result of lesser interaction between cells as the activity of hPSCs was inhibited by FGF2. As suspected, the ATP content of the heterospheroids (**Figure 6G**) indicated less activity in the FGF2 and FGF2-SPION treated heterospheroids. In the other setting, treatment with gemcitabine alone reduced the growth rate of spheroids as indicated by the reduced spheroid size and cell debris in the surrounding, while the co-treatment with FGF2 or FGF2-SPIONs resulted in higher reduction in the spheroid size (**Figure 6E-F**). In contrast, combination of SPIONs with gemcitabine did not show any significant effect. As shown in **Figure 6G**, co-treatment of FGF2 or FGF2-SPIONs with gemcitabine led to a significant reduction in the ATP content compared to gemcitabine alone. Furthermore, we investigated whether the treatment with FGF2-SPIONs also leads to the inhibition of tumor stroma by attenuating the activation of hPSCs. We therefore examined the expression of α-SMA in the treated heterospheroids and found that FGF2-SPIONs significantly reduced the expression of α-SMA in spheroids without or with gemcitabine treatment (**Figure 6H-I**).

**Figure 7:**
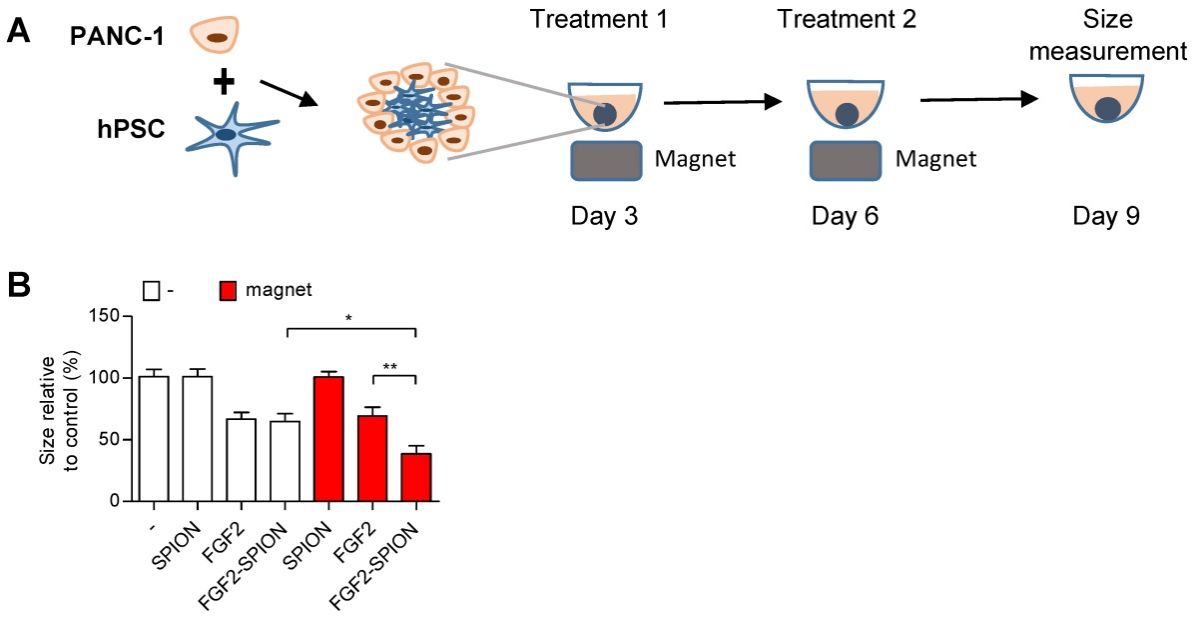
** Magnetic driven iron oxide accumulation.** Schematic representation (A) of spheroid culture in 96 round-bottom well plate with neodymium magnet as driving force to attract SPION or FGF2-SPION. Measured size (B) of spheroids after 9 days in culture. Data represents mean + SEM for at least 8 spheroids. Statistical differences are *p<0.05, **p<0.01.

One of the applications of SPIONs is to guide them to the tumor site using external magnetic field 33, 34. To demonstrate the applicability of FGF2-SPIONs in this direction, we set up an experiment in which we applied an external magnetic field using a neodynium magnet underneath the cell culture plate wells, as illustrated in **Figure 7A**. Interestingly, the application of magnetic field led to the induced effect of FGF2-SPIONs by reducing the spheroids size compared to the ones treated with FGF2-SPIONs without the magnetic field (**Figure 7B**). There was no additional effect of the magnetic field on FGF2 or SPIONs treated spheroids.

## Discussion

In the present study, we demonstrate for the first time that targeting of FGF2 using SPIONs reduces the tumor stroma and potentiates the effect of chemotherapy in a stroma-rich 3D spheroid model. Tumor stroma plays a crucial role in the induction of tumor growth and versatile adversity in tumor progression as well as acts as a barrier to chemotherapy 10, 35. Inhibition of tumor stroma is therefore an interesting approach to reduce stroma-induced tumor growth as well as to enhance the effect of chemotherapy. We engineered SPIONs by conjugating with FGF2, a growth factor known to counteract TGF-β effect. We showed that FGF2-SPIONs could inhibit TGF-β differentiation of hPSCs into myofibroblastic CAFs and CAFs-induced ECM production. Furthermore, in stroma-rich 3D heterospheroids, we demonstrated that FGF2-SPION were highly effective in reducing stroma-induced tumor spheroid growth. This inhibitory effect of FGF2-SPIONs was further enhanced in the presence of the external magnetic field.

FGF2 is an interesting growth factor, which is known to inhibit fibroblasts activation *in vitro* by distinct signaling pathways such as Smad2/3 and MAPK pathways 15, 16, 18. The differentiation and activation of hPSCs into CAF-like myofibroblasts is mainly induced by TGF-β as a result of the interaction with cancer cells within the tumor microenvironment. Although FGF2 binds to four different FGF receptors, in the present study, we showed that hPSCs only overexpressed FGFR3c in response of TGF-β and this receptor has been shown to have high binding specificity and activity with FGF2 36, 37. Inhibition of TGF-β-induced hPSCs differentiation by FGF2 was attributed to the inhibition of Smad2 phosphorylation. Over-activation of pro-mitogenic pathways such as TGF-β dependent cytokine expression (EGF, FGF, HGF, etc.) may modify TGF-β response 38. Active FGF2 signalling activates downstream mediators Ras and phosphoinositide 3-kinase (PI3K) and results in inhibition of Smad2/3 phosphorylation 14, 38. CAFs are the main component to produce abundant ECM, which together with high contractility of CAFs, can build up dense matrix and increase intratumoral tension, resulting in poor penetration of therapeutics 39. Reduction of fibrosis has been shown to enhance the therapeutic efficacy of chemotherapy in PDAC tumors, as shown by us and others 21, 40-42. In the present study, treatment with FGF2 reduced the TGF-β-induced collagen production as well as contraction* in vitro*, which shows its ability to inhibit key stromal features in relation to the stromal barrier.

Biologics such as peptides and proteins are highly potent natural or engineered therapeutics but are prone to enzymatic degradation and are rapidly eliminated from the body. To protect them from the degradation and prolong their systemic circulation, various types of nanocarriers are developed and commonly used 43. In the present study, we utilized SPIONs to target FGF2 to tumor stroma, as benefits of using SPIONs are manifold, including large surface area and surface functionalization allowing easy conjugation of ligands/proteins on the surface, small size evading reticuloendothelial system and better tumor penetration 44, 45, improved targeting efficiency using external magnetic field and detection by MRI 46. Earlier, we have shown that targeting of relaxin hormone, a sensitive biologic, using SPIONs could induce its effect *in vivo* in pancreatic tumor model in mice 21. In the present study, conjugation of FGF2 on the surface of SPIONs was successfully achievable without the loss of its bioactivity. FGF2 has an isoelectric point of 9.2 and at pH 7 the net charge will be about positive. However, during conjugation of FGF2 to SPION, we use several amine groups (responsible for positive charge of FGF2) of FGF2, which will shift the net charge on FGF2 towards the negative side, hence on FGF2-SPION. Furthermore, to show specificity of FGF2-SPION to FGFR3c (only FGFR overexpressed on TGF-β-activated hPSCs), we found that FGF2-SPION showed strong binding (6-fold) to TGF-β-activated hPSCs than non-activated hPSCs and there was no binding of SPION alone to activated and non-activated PSCs. These data indicate that It is also noteworthy that after conjugation such a protein can lose its activity due to modification at the receptor interaction site or steric hindrance caused by the conjugation. However, our *in vitro* studies showed that FGF2-SPIONs did not lose the biological activity, as can be seen in Figure 4B-C. Interestingly, FGF2-SPIONs retained the inhibitory effects of FGF2 on the hPSC activation at higher concentrations at which free FGF2 showed the contrasting effect. Such biphasic effects of FGF2 have been reported earlier 29-32. Garcia-Maya and co-workers showed that low concentrations of FGF2 inhibit proliferation while intermediate concentrations stimulate proliferation in the presence of serum. Intriguingly, high concentrations reverse the proliferation effects, and mirror the low concentration effects: inhibition of proliferation and stimulation of survival and differentiation. They show that the peak in proliferation correlates with abrupt activation of FRS-2 and Erk pathways that is specifically down-regulated by high concentrations of FGF2 30. In our study, we observed the similar biphasic effects of free FGF2 i.e. inhibition at 250 ng/ml and reversal effect at 500 ng/ml. In contrast, FGF2-SPION showed only inhibitory effects at both 250 ng/ml and 500 ng/ml but the effects at 500 ng/ml were not higher than 250 ng/ml. This might be due to limited exposure of FGF2 to the receptor when bound to SPION, likely due to steric hindrance thus allowing inhibitory effects both at 250 or 500 ng/ml but no reversal effects. This provides a real benefit to our approach to gain only inhibitory effects.

In the complex tumor microenvironment, PSCs interact with tumor cells and induce their growth, not only by secreting cytokines and growth factors, but also by establishing physical contacts. These interactions pose barriers to nanotherapy penetration and efficacy *in vivo* 47. 3D culture models such as tumor spheroids with the stroma component mimic the *in vivo* tumor and represent more realistic recapitulation of the tumor microenvironment 48, 49. To mimic the tumor stroma in 3D culture, in this study, we generated self-assembled 3D heterospheroids of PANC-1 and hPSC and demonstrated that co-culturing of tumor cells with hPSCs induces the tumor spheroid growth. These data are in line of our previous studies performed in 2D and 3D cultures 50, 51. In the present study, we found the enhanced efficacy of gemcitabine when combined with FGF2 or FGF2-SPIONs which was attributed to the reduced activation of hPSCs as confirmed with the reduction of α-SMA, the fibroblast activation marker. Magnetic field-guided targeting of SPIONs towards the tumor site is one of the main applications of SPION 33, 34. Our data showing the enhanced effect of FGF2-SPIONs on 3D spheroid size with the external magnetic field clearly indicate the benefit of targeting FGF2 using SPIONs.

In conclusion, this study demonstrates a novel strategy to target FGF2 to tumor stroma using SPIONs and thereby induce the efficacy of gemcitabine by reducing the stroma barrier. Targeted delivery using SPIONs can potentially be exploited for other biologics to enhance their therapeutic efficacy with opportunities for diagnostics and magnetic field guided applications.

## Figures and Tables

**Table 1:** Sequences of forward and reverse primers used during real-time PCR.

Gene	Forward Primer	Reverse Primer
r18s rRNA	TGAGGTGGAACGTGTGATCA	CCTCTATGGGCCCGAATCTT
Acta-2	CCCCATCTATGAGGGCTATG	CAGTGGCCATCTCATTTTCA
Collagen1α1	GTACTGGATTGACCCCAACC	CGCCATACTCGAACTGGAAT
FGFR1c	GGACTCTCCCATCACTCTGCAT	GGCCCCTGTGCAATAGATGA
FGFR2b	ACAGCTTCCCCAGACTACCT	CAGGGGGATACGTTTGGTCA
FGFR2c	GCCAAGCCTGAGTCCTTTCT	ACGCAGAAGAGTGGTCCTTG
FGFR3b	CGACGAGTACCTGGACCTGT	CCTCACATTGTTGGGGACCA
FGFR3c	GACGTACACGCTGGACGTGCTGGA	AGCACCACCAGCCACGCAGAGTGA
FGFR4	AGTTCTGCCTACAGGACACG	ACAGGAGTCCCACCGTGTAT

**Table 2:** Details of the antibodies used in the study.

Antibody	Source	Dilution	
		Blotting	IFC
Rabbit monoclonal anti-bFGF	Cell Signaling	1:1000	
Goat anti-type I collagen	Southern Biotech	1:300	1:300
Mouse monoclonal anti-actin, α-smooth muscle	Sigma Aldrich	1:600	1:600
Rabbit monoclonal anti-phospho-Smad2/3	Cell Signaling	1:1000	
Rabbit monoclonal anti-Smad2/3	Cell Signaling	1:1000	
Rabbit monoclonal anti-phospho-ERK1/2	Cell Signaling	1:1000	
Mouse monoclonal anti-ERK1/2	Cell Signaling	1:1000	
Mouse monoclonal anti-β-actin	Sigma Aldrich	1:5000	
Polyclonal rabbit anti-goat immunoglobulin HRP	Dako	1:2000	
Polyclonal goat anti-rabbit immunoglobulin HRP	Dako	1:2000	
Polyclonal goat anti-mouse immunoglobulin HRP	Dako	1:2000	
Polyclonal rabbit anti-mouse immunoglobulin HRP	Dako	1:2000	
Alexa Fluor® 488 donkey anti-mouse IgG	Invitrogen		1:100
Alexa Fluor® 594 donkey anti-goat IgG	Invitrogen		1:100

## Abbreviations

α-SMA: α-smooth muscle actin
CAFs: cancer-associated fibroblasts
CM: Conditioned Medium
Col-I: collagen-I
ECM: extracellular matrix
FGF2: fibroblast growth factor 2
hPSCs: human pancreatic stellate cells
PDAC: pancreatic ductal adenocarcinoma
SPIONs: superparamagnetic iron oxide nanoparticles
TGF-β: transforming growth factor β
